# Conserved specificity of extracellular wastewater peptidases revealed by multiplex substrate profiling by mass spectrometry

**DOI:** 10.1007/s10311-025-01834-7

**Published:** 2025-04-12

**Authors:** Natalie Wichmann, Josephine Meibom, Tamar Kohn, Michael Zumstein

**Affiliations:** 1 Division of Environmental Geosciences, Centre for Microbiology and Environmental Systems Science, University of Vienna, Josef-Holaubek-Platz 2, 1090 Vienna, Austria; 2 Department of Environmental Microbiology, Swiss Federal Institute of Aquatic Science and Technology (Eawag), Überlandstrasse 133, 8600 Dübendorf, Switzerland; 3 Laboratory of Environmental Virology, School of Architecture, Civil and Environmental Engineering, École Polytechnique Fédérale de Lausanne (EPFL), Station 2, 1015 Lausanne, Switzerland

**Keywords:** Peptidase specificity, Wastewater systems, Multiplex substrate profiling by mass spectrometry, Peptide-based chemicals, Environmental fate, Benign-by-design

## Abstract

Peptide-based chemicals are promising for numerous applications including home and personal care and medical treatments. To better understand and control the environmental fate of peptide-based chemicals, in-depth knowledge on the specificity of wastewater peptidases is needed. Here, we employed multiplex substrate profiling by mass spectrometry to obtain specificity profiles of extracellular peptidases derived from influent and aeration tanks of three full-scale wastewater treatment plants. Specificities were confirmed by fluorogenic peptidase substrates. Our results revealed highly similar specificity profiles across wastewater treatment plants. We found that hydrolysis by extracellular wastewater peptidases is favored when positively charged amino acid residues surround the cleavage site and disfavored when negatively charged amino acid residues surround the cleavage site.

**Supplementary Information:**

The online version contains supplementary material available at 10.1007/s10311-025-01834-7.

## Introduction

Peptide-based chemicals are increasingly used in home and personal care products [1–3] and as therapeutics [4–6]—mainly due to their effectiveness, versatility, and selectivity. After use, a substantial fraction of chemicals used in these applications enters wastewater streams and—when incompletely removed during treatment—natural environments [7–9]. Previous studies revealed that wastewater systems harbor an array of active peptidases [10–12]. If the specificity of wastewater peptidases matches the chemistry of peptide-based chemicals, peptidase-mediated breakdown can occur—with the potential to mitigate adverse effects on wastewater-receiving environments. [7,13,14]

However, the current understanding of the activity and specificity of peptidases in wastewater systems is limited. Notably, substantial (extracellular) peptidase activity has been reported not only in bioreactors (e.g., aeration tanks) of wastewater treatment plants (WWTPs), but also in WWTP influents (i.e., untreated wastewater) [11,13,14,16]. Previous studies indicated that peptides larger than ~ 650 Da are unlikely to be transported across microbial membranes and that extracellular hydrolysis is thus a crucial step in the degradation of peptide-based chemicals [17,18]. While extracellular peptidases in influent samples were predominantly found in the dissolved fraction, the predominant extracellular peptidase activity in aeration tank samples was detected in the fraction bound to the extracellular polymeric substance (EPS) [14]. A recent study on the biotransformation of antimicrobial peptides suggested that extracellular influent peptidases exhibited substantial specificity and that this specificity was largely conserved across the tested WWTPs [14]. However, the tested set of peptides covered only a small fraction of all possible amino acid combinations. In another study, the specificities of extracellular peptidases in aeration tank extracts were assessed with a peptide library derived from a model protein (i.e., bovine serum albumin) [16]. A limited number of detected hydrolyses and similar patterns across WWTPs were found, again suggesting substantial and conserved peptidase specificity. However, only 40% of all possible amino acid pairs were covered by the model protein-derived peptide library and trypsin-like specificities could not be assessed due to the use of trypsin when creating the library. A comprehensive assessment of the specificity of extracellular wastewater peptidases is therefore lacking.

An approach that enables such a comprehensive peptidase specificity assessment is multiplex substrate profiling by mass spectrometry (MSP-MS). This approach, developed for peptidases by O’Donoghue and colleagues,^19^ employs a systematically designed library of model peptide tetradecamers. After incubating the library with peptidases, liquid chromatography coupled to high-resolution mass spectrometry (LC-HRMS) is used to detect hydrolysis products. From these data, a comprehensive peptidase specificity profile—representing the amino acid frequency surrounding the detected hydrolysis sites relative to their frequency in the library—is generated. Because MSP-MS offers an in-depth view on peptidase specificity, it has been utilized to study both isolated peptidases,^19,20^ as well as mixtures of peptidases; however, it has not been applied to wastewater systems [19,21,22]. While MSP-MS has also been established for other enzyme classes that may be relevant in wastewater (e.g., lipases [23] or glycosidases [24]), we herein focused on peptidases with the potential of hydrolyzing peptide-based chemicals.

Here we mapped the specificity of extracellular peptidases in influent and aeration tanks of full-scale municipal WWTPs. Therefore, we extracted extracellular peptidases from samples taken at three WWTPs and employed the MSP-MS approach. To orthogonally validate the resulting peptidase specificity profiles, we investigated enzyme kinetics of wastewater extracts with selected fluorogenic peptidase substrates. By revealing specificity profiles of extracellular peptidases in wastewater systems, our results can improve fate predictions of peptide-based chemicals and inform the design of peptide-based chemicals that are rapidly broken down in wastewater systems.

## Experimental

Detailed information on chemicals and materials, total suspended solids, protein concentration, and peptidase activity determinations, ultra high performance liquid chromatography coupled to high resolution mass spectrometry (UHPLC-HRMS/MS) measurements, and data processing is provided in the Supporting Information (SI).

### Wastewater sampling and enzyme extraction

Wastewater samples were obtained at three municipal WWTPs in Austria at dry weather conditions (i.e., no precipitation on the sampling day and the day before). Information on WWTPs is provided in Table S1. We took 0.7 L grab samples from influents and aeration tanks using a plastic beaker and transferred them into 1-L glass bottles (leaving 300 mL headspace) for transport to the laboratory; 24-h composite influent samples were obtained from WWTP A using an autosampler collecting 30 mL per 40 m^3^ of influent load (stored at 4 °C until collection after 24 h). Enzyme extraction started within one hour after sampling. Influent extracts were prepared by centrifugation of 20 mL of raw wastewater (10 min, 2′000 g) and sterile filtration (0.2 µm PES, Millipore Stericup, S2GPU05R). Aeration tank extracts were prepared by adding cation exchange resin (10% (w/v), Sigma-Aldrich, 91,973) to 20 mL of wastewater sample (to release EPS-bound enzymes [25,26]), followed by horizontal shaking (30 min, 125 rpm, room temperature) and centrifugation and sterile filtration as described above for influent extracts. [13,14]

### Peptide library incubations with extracted enzymes

Incubations were performed at room temperature using a final peptide concentration of 0.5 µM per peptide and a final reaction volume of 400 µL. At defined time points (i.e., 0.01, 0.5, 1, 2, and 4 h) of incubation after spiking the library, 40 µL of solution was transferred to a new Protein LoBind tube and incubated at 80 °C for 10 min. For the initial sampling time point, the time between spiking and transferring samples for heat inactivation was less than 30 s and inactivation of peptidases at 80 °C was assumed to occur within 5 min of incubation; therefore, the sample is referred to as t = 5 min). Subsequently, the tubes were centrifuged for 1 min at 20′817 *g* at room temperature and the supernatant was transferred to a clean LC–MS vial. Samples were stored at -20 °C until UHPLC-HRMS measurements. For abiotic control incubations, wastewater extracts (3 × 1.8 mL) were autoclaved (20 min, 121 °C, 2 bar, Wolf Sanoclav LaS-MCS-J). Peptide calibrations were prepared using ultrapure water as well as the respective autoclaved wastewater extract spiked at equimolar peptide concentrations ranging from 0.001 µM to 0.5 µM.

For α-chymotrypsin incubations, we used an enzyme-to-peptide ratio of 1:100 and a final reaction volume of 250 µL. Incubations were conducted at 37 °C using active and autoclaved α-chymotrypsin solution as described above. At defined time points (i.e., 0.01, 1, 4 and 24 h), 40 µL of the solution were transferred to a new Protein LoBind tube and reaction quenching and sample preparation were performed as described above.

### Liquid chromatography and mass spectrometry

A detailed description is provided in the SI. In brief, we analyzed peptides using UHPLC (Thermo Scientific Vanquish Horizon) equipped with a Waters XSelect PREMIER CSH C18 column coupled to HRMS (Thermo Exploris 240). For data processing, we performed label-free quantification and database search against the peptide sequences of the peptide library using Peaks Studio 11 (bioinformatics Solutions Inc.), followed by removal of peptides with low quality scores and data normalization. To consider all products of hydrolytic reactions, we did not employ a signal intensity threshold and transformation products were defined as products having an intensity score of ≥ 8 compared to the autoclaved sample after the same incubation period as the respective active sample (Figure S1).

### Peptidase activity measurements with fluorogenic substrates

To each well of a black 96-well microplate (Millipore-Sigma, CLS3991), we added 50 μL enzyme extract, 100 μL of the respective fluorogenic substrate or 7-amido-4-methylcoumarine (AMC) standard, and 50 μL Tris–HCl buffer (50 mM, pH 7.8) and monitored fluorescence using a Tecan Infinite 200 pro plate reader (excitation: 360 nm, emission: 450 nm, reading interval: 4 min, measurement duration: 60 min). Concentration ranges were 0.5–10 µM for all substrates. We used matrix-matched calibrations with AMC concentrations ranging from 0.5 to 10 µM and confirmed the absence of substrate hydrolysis in autoclaved wastewater extract and ultrapure water.

## Results and discussion

### Optimization of multiplex substrate profiling by mass spectrometry

For method optimization, we assessed peptide recoveries from ultrapure water across different quenching protocols. In addition to the original protocol, i.e., protein denaturation in urea followed by solid-phase extraction using C18 ZipTips [19,27], we tested quenching enzymatic processes by using acetonitrile containing 1% formic acid or by heating the samples to either 70 °C, 80 °C or 90 °C for 10 min (Figure S2-S3). While recoveries for the original protocol were low (Figure S2), the heat inactivation protocol at 80 °C resulted in recoveries exceeding 75% for all peptides (**Figure S3**). Because this protocol also sufficiently inactivated enzymes (confirmed by consistent peptide concentrations in different replicate samples measured at 6-h intervals), we chose this protocol for all further analyses. We validated this adapted multiplex substrate profiling by mass spectrometry (MSP-MS) workflow by confirming the specificity of α-chymotrypsin for bulky aromatic amino acids (i.e., tyrosine, phenylalanine, and tryptophan) in P1 position (Figure S4). [28]

### Extracellular wastewater extracts

To assess the specificity of extracellular wastewater peptidases, we applied the adapted MSP-MS approach to extracellular dissolved wastewater extracts from influents and extracellular dissolved and extracellular polymeric substances (EPS)-bound extracts from aeration tank extracts [14]. Peptidase activity, protein concentration, and total suspended solids content were consistent with previous studies at the same wastewater treatment plants (WWTPs) (Figure S5**)**. [14] We confirmed the absence of library peptides in wastewater extracts, as well as the absence of abiotic formations of library peptide hydrolysis products, with abiotic control incubations. These control experiments included spiking the pooled peptide library to ultrapure water and autoclaved wastewater extract. We ascribed the minor concentration differences between the nominally spiked concentration and the initially detected concentration of some peptides in ultrapure water and autoclaved wastewater extract to losses during sample preparation. Differences in peptide concentrations between active and autoclaved samples at the initial sampling time point were ascribed to either rapid enzymatic peptide hydrolysis or different sorption properties due to autoclaving. A decrease in peptide concentration over time in active wastewater samples, but not autoclaved samples, was ascribed to enzymatic peptide hydrolysis. We note that the chosen incubation period of four hours is in the range of hydraulic retention times of most sewage systems and WWTP aeration tanks (Table S1). The observed rapid peptide hydrolysis highlights the potential of leveraging wastewater peptidases for the breakdown of peptide-based chemicals.

**Fig. 1:**
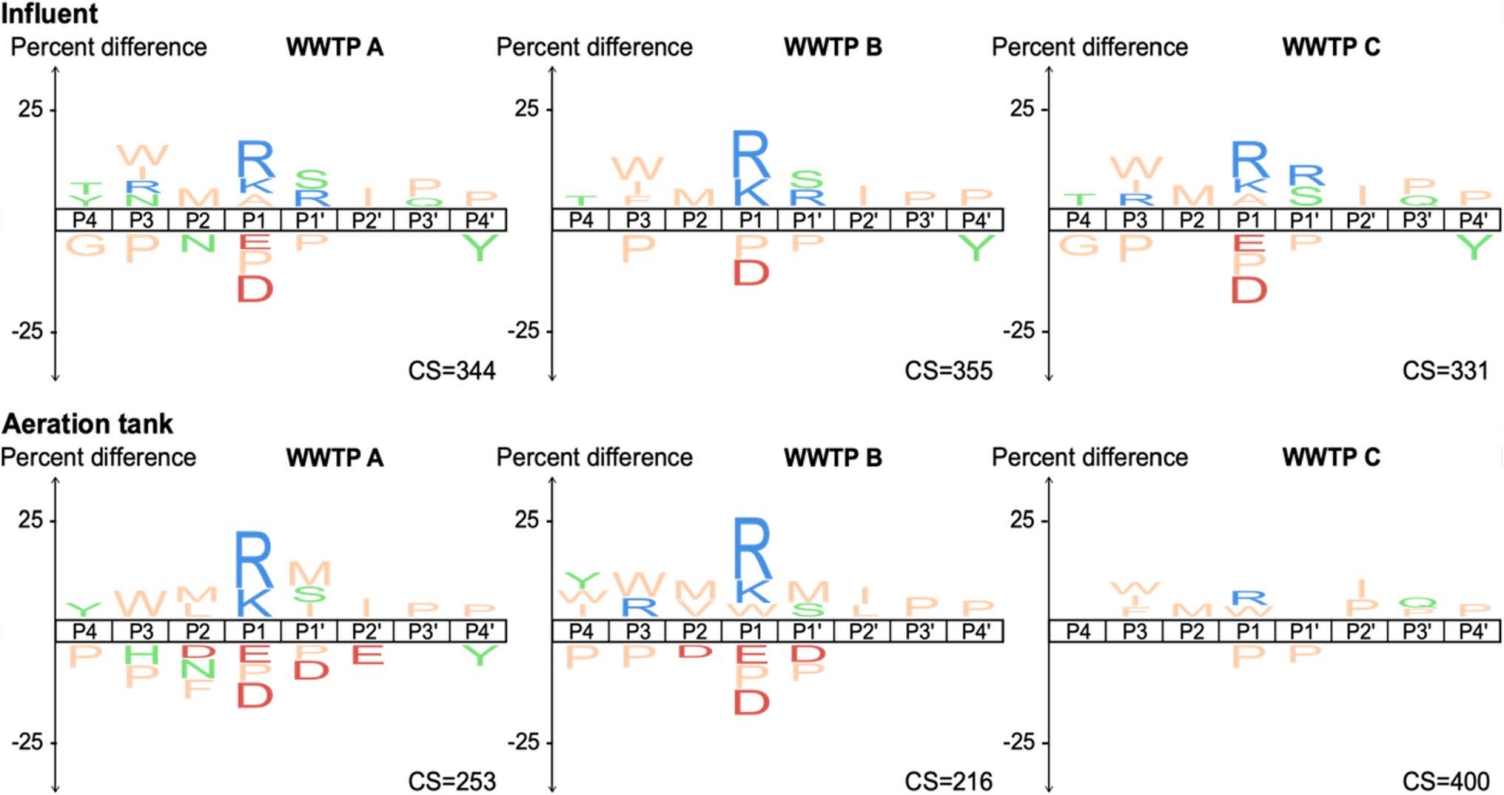
Specificity profiles of incubations with extracellular extracts from influents and aeration tanks of three wastewater treatment plants (WWTPs) at the initial sampling time point (t = 5 min). IceLogos represent the amino acid frequency surrounding the detected hydrolysis sites relative to their frequency in the library. One letter amino acid code is used (with M representing norleucine) according to the following color code: positively charged in blue, negatively charged in red, polar in green, and nonpolar in orange (charge state defined at pH = 7). The four amino acid residues located N-terminally and C-terminally of the hydrolysis site are referred to as P4-P1 and P1’-P4’, respectively. CS: number of detected cleavage sites

Analyzing the resulting transformation products revealed approximately 300 cleavage sites at the initial sampling time point in both influent extracts and aeration tank extracts (Fig. 1). Diluting enzyme extracts 1:100 with autoclaved extract reduced the number of cleavage sites after 4 h of incubation by over 95%, confirming that the observed high numbers of cleavages in non-diluted extracts resulted from rapid peptide hydrolysis (Figure S6). With respect to peptidase specificity in influent and aeration tank samples, we consistently—and across all tested WWTPs—observed a preference for hydrolysis when the basic amino acids lysine and arginine were in P1 position. Conversely, hydrolysis was disfavored when negatively charged amino acids, such as aspartic acid and glutamic acid, occupied the P1 position (Fig. 1, S7-S8). These findings were consistent with a recent study on the fate of antimicrobial peptides in wastewater systems, where hydrolysis of positively charged amino acids in P1 position was reported, but none of the detected products resulted from hydrolysis of peptide bonds with negatively charged amino acid residues in P1 position [14]. These observations might indicate that the binding pocket of the predominant peptidases in wastewater is partially negatively charged. Therefore, incorporating a positively charged amino acid into the structure of a peptide-based chemical during its (re-)design might enhance its biotransformation rate in wastewater systems. The previous study using a model protein-derived peptide library to assess extracellular peptidase specificities in aeration tanks did not capture the herein detected specificity due to the similarity to the specificity of trypsin, which was used to create the library [16]. We further observed that hydrolysis was consistently disfavored when proline occupied the P1 or P1’ position, which is likely caused by the enhanced rigidity proline confers to peptides [29]. While MSP-MS provides comprehensive insights into peptidase specificities, we acknowledge certain limitations of this experimental approach. The use of presumably linear peptides does not account for secondary or tertiary structural effects on hydrolysis. The specificity profiles might further be influenced by the used extraction method, possibly missing certain peptidases. Lastly, certain hydrolysis products might not be detectable by the analytical technique (e.g., due to very small size, high polarities, or poor ionization efficiencies).

The similarity between extracellular peptidase specificities in influent and aeration tank samples suggests two possibilities. Either the majority of peptidases in aeration tanks originate from the influent and—upon entering the aeration tank—bind to the EPS, or the microbes in the aeration tank secrete peptidases with specificities similar to those found in the influent. To draw conclusions, a deeper understanding of the origin and the identity of these peptidases is required. Gaining insights into the identity of extracellular peptidases will furthermore reveal information on the number and diversity of the wastewater peptidases that result in the activity and specificity reported here. Extracts from WWTP C aeration tanks exhibited less specificity compared to the other extracts, which is also reflected by the higher number of cleavage sites (Fig. 1). For all extracts, the number of cleavage sites increased over time and hence the observed specificity profiles became less pronounced (Figure S7). By additionally analyzing a 24-h composite influent sample and an additional aeration tank grab sample from WWTP A, we found that the specificity profiles were also conserved across these sampling days (Figure S7**)**. To quantitatively assess the observed similarities, we calculated pairwise Pearson correlation coefficients (r) of amino acid frequencies in the hydrolyzed sequences between sampling days (r > 0.9), WWTPs (r > 0.9, except when aeration tank of WWTP C was involved, see above), and between influent and aeration tank of a WWTP (0.6 < r < 0.9) (Table S2). These calculations highlighted the high similarity between peptidase specificities across WWTPs and sampling dates, which is in agreement with previous studies. ^14,16^

### Validating wastewater peptidase specificity with fluorogenic substrates

**Fig. 2:**
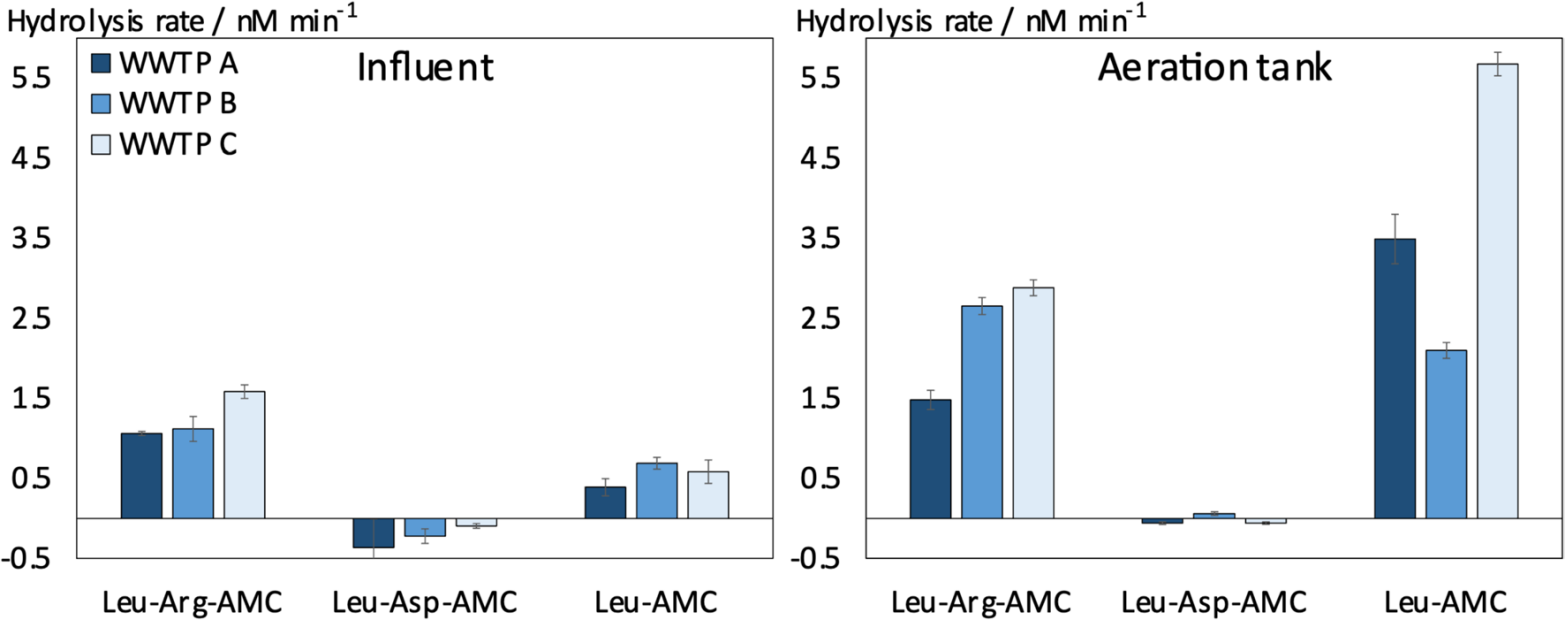
Hydrolysis rates of fluorogenic probes in wastewater extracts. For influent incubations, extracellular dissolved extracts and for aeration tanks, extracellular dissolved and extracellular polymeric substance-bound extracts from three different full-scale wastewater treatment plants (WWTPs) were used. Substrate concentration: 1 µM. Peptidase substrates represented in three-letter amino acid code: leucyl-arginine-4-methylcoumaryl-7-amide (Leu-Arg-AMC), Leucyl-aspartic acid-4-methylcoumaryl-7-amide (Leu-Asp-AMC), and leucine-7-amido-4-methylcoumarine (Leu-AMC). Data points and error bars represent mean ± standard deviation of triplicate incubations

To confirm the most distinct MSP-MS results, i.e., favored hydrolysis when arginine occupied the P1 position and disfavored hydrolysis when aspartic acid occupied the P1 position, we selected two fluorogenic substrates (i.e., leucyl-arginine-4-methylcoumaryl-7-amide (Leu-Arg-AMC) and leucyl-aspartic acid-4-methylcoumaryl-7-amide (Leu-Asp-AMC)). Incubating these substrates with wastewater peptidases at similar concentrations as used in the MSP-MS assay revealed that Leu-Arg-AMC is readily hydrolyzed, whereas Leu-Asp-AMC is not hydrolyzed by wastewater peptidases, which is in agreement with the MSP-MS results (Fig. 2). An exemplary substrate concentration-dependent increase in fluorescence intensity during the incubation of a fluorogenic probe with wastewater peptidases, as well as the complete collected kinetic data (i.e., concentration-dependent hydrolysis rates), are shown in Figures S9 and S10. For Leu-Arg-AMC, we consistently found slightly lower hydrolysis rates in influent extracts compared to aeration tank extracts for all tested WWTPs. For Leu-Asp-AMC, hydrolysis rates were low for influent as well as aeration tank extracts, which we ascribed to the lack of specificity of the peptidases for this substrate (Fig. 2). To test whether the hydrolysis of Leu-Arg-AMC is catalyzed by endo-acting peptidases (rather than consisting of two subsequent exo-type hydrolyses), we additionally derived hydrolysis kinetics for leucine-7-amido-4-methylcoumarine (Leu-AMC). For influent extracts, these tests showed lower hydrolysis rates compared to Leu-Arg-AMC and thus confirmed endo-type hydrolysis. Conversely, in aeration tank extracts, reaction rates using Leu-AMC were similar (WWTP B) or even higher (WWTPs A and C) compared to Leu-Arg-AMC, indicating that exo-type hydrolyses might substantially contribute to the detected hydrolysis of Leu-Arg-AMC hydrolysis (Fig. 2).

## Conclusion

Our study highlights the potential of extracellular peptidases in wastewater systems to hydrolyze anthropogenic peptide-based chemicals. We report a distinct specificity pattern for these peptidases, i.e., preferential hydrolysis of peptide bonds located C-terminally of positively charged amino acid residues, and show that these patterns were largely conserved across the three tested WWTPs as well as between extracts from influent and aeration tanks. Our findings raise fundamental questions regarding the origin of these peptidases, their global distribution in wastewater systems, and how their activity and specificity is affected by variable wastewater conditions—which remain to be addressed in future work. A comprehensive understanding of wastewater peptidases will improve fate predictions of peptide-based chemicals and inform the design of peptide-based chemicals for rapid biodegradation by wastewater microbiomes. We note that structural aspects of peptide-based chemicals that were not studied here and remain to be investigated can also affect hydrolysis rates of such motives. Additionally, the effect of incorporating such motives on the properties and (shelf-)stability needed for the respective application of a peptide-based chemical need to be assessed, as well as the environmental fate and effects of hydrolysis products of peptide-based chemicals.

## Supplementary Information

Below is the link to the electronic supplementary material.

Supplementary file1 (DOCX 22912 kb)

## Data Availability

Data will be made available on request.

## Code availability

Code will be made available on request.
