# Supplementary material for: Conserved specificity of extracellular wastewater peptidases revealed by multiplex substrate profiling by mass spectrometry
Source: Environ Chem Lett. 2025 Apr 12;23(4):953–9. doi: 10.1007/s10311-025-01834-7 (PMC12119764; doi:10.1007/s10311-025-01834-7)
Supplement: Supplementary file 1 — Supplementary file1 (DOCX 22912 kb) [file 10311_2025_1834_MOESM1_ESM.docx]

*Supplementary Material*

**Conserved specificity of extracellular wastewater peptidases revealed by multiplex substrate profiling by mass spectrometry**

Natalie Wichmann^1,2^, Josephine Meibom^3^, Tamar Kohn^3^ and Michael Zumstein^1*^

^1^Division of Environmental Geosciences, Centre for Microbiology and Environmental Systems Science, University of Vienna, Josef-Holaubek-Platz 2, 1090 Vienna, Austria

^2^Department of Environmental Microbiology, Swiss Federal Institute of Aquatic Science and Technology (Eawag), Überlandstrasse 133, 8600 Dübendorf, Switzerland

^3^Laboratory of Environmental Virology, School of Architecture, Civil and Environmental Engineering, École Polytechnique Fédérale de Lausanne (EPFL), Station 2, 1015 Lausanne, Switzerland

^*^To whom correspondence should be addressed:

E-mail: michael.zumstein@univie.ac.at

**CONTENT**

**Materials and Methods**

**Table S1.** Wastewater treatment plant (WWTP) parameters on sampling days.

**Table S2.** Pearson Correlation Coefficients of amino acid frequencies surrounding the cleavage sites of library peptides after hydrolysis by peptidases from different full-scale wastewater treatment plants (WWTPs).

**Figure S1.** Effect of different parameter thresholds of multiplex substrate profiling (MSP-MS) workflow on the specificity profiles.

**Figure S2.** Comparison of peptide recoveries using different protocols to stop enzymatic activity.

**Figure S3.** Comparison of peptide recoveries using different temperatures to stop enzymatic activity.

**Figure S4.** Multiplex substrate profiling by mass spectrometry workflow validation using α-chymotrypsin.

**Figure S5.** Peptidase activity and protein concentration of wastewater extracts and total suspended solid concentrations of aeration tank grab samples from three different wastewater treatment plants (WWTPs).

**Figure S6.** Specificity profiles of incubations using diluted and non-diluted wastewater extracts with library peptides.

**Figure S7.** Specificity profiles of incubations using wastewater extracts with library peptides.

**Figure S8**. Hydrolyzed amino acid pairs during the incubation of wastewater extracts with the library peptides.

**Figure S9.** Increase in fluorescence intensity upon hydrolysis of a fluorogenic probe by wastewater peptidases.

**Figure S10.** Michaelis-Menten reaction kinetics using wastewater extracts incubated with different fluorogenic substrates.

**Supplementary Materials and Methods**

**Chemicals and Materials**

All solutions were prepared using ultrapure water (ELGA PURELAB Pharma Compliance, 0.075 µS, pH 6.8). Acetonitrile (hypergrade for LC-MS LiChrosolve, Merck, ≥ 99.9 %, 100292500) was purchased from VWR. Formic acid (98 - 100 %, 5.43804) and tris(hydroxymethyl)aminomethane (Tris, ≥ 99.8 %, 252859) were obtained from Sigma-Aldrich. Safe-Lock Tubes (0.5, 1.5, and 2 mL), Protein LoBind tubes (1.5 mL (0030108116) and 2 mL (0030108132)), and epT.I.P.S (0.1-20 µL and 2-200 µL) were purchased from Eppendorf. 50 - 1000 µL Universal blue Tips were purchased from VWR.

All peptides of the peptide library^1^ were custom-synthesized by the University of Lausanne or by GenScript Biotech. The library contains 124 tetradecameric peptides, which contain two duplicates of each amino acid pair (XY). Additionally, the library contains one instance of every X*Y and X**Y pair, with X and Y denoting specific amino acids and * representing randomly assigned amino acids, positioned at the center of the sequence. Distinct dipeptides at both termini of the peptides enable the identification of exocleaving peptidase specificities, resulting in a total of 1612 cleavage sites within the library.^1^ Peptide stock solutions were prepared from dried peptides at 20 mM in dry DMSO. From these solutions, each peptide was diluted further to yield 5 mM stock solutions in DMSO in Protein LoBind tubes. 5 µL of each peptide were combined in a new Protein LoBind tube to yield the pooled peptide library containing all 124 peptides at a final concentration of 40.32 µM. Aliquots of the pooled peptide library were stored at -20 °C until further use.

Aqueous α-chymotrypsin (bovine pancreas, type II, ≥ 85 %, C4129, Sigma Aldrich) was prepared at a concentration of 20 µg/mL and aliquots were stored at -20 °C until further use.

7-amido-4-methylcoumarine (AMC, 99 %, 257370, Sigma Aldrich) stock solution was prepared at a final concentration of 5 mM in aqueous methanol (50 % (v/v)). Substrate stock solutions (L-Leucine-7-amido-4-methylcoumarine (Leu-AMC, ≥ 99.7 %, HAA3200, Iris Biotech), Leucyl-aspartic acid-4-methylcoumaryl-7-amide (Leu-Asp-AMC, ≥ 99 %, 3235-v, PeptaNova), and Leucyl-arginine-4-methylcoumaryl-7-amide (Leu-Arg-AMC, ≥ 95 %, GenScript Biotech)) were prepared in aqueous methanol (50 % (v/v)) at a final concentration of 10 mM. Working solutions were freshly prepared by diluting the stock solution with ultrapure water.

**Total Suspended Solids, Protein Concentration and Peptidase Activity Determination**

*Total suspended solid* (TSS) *determination*. A vacuum filtration system was used to filter aeration tank samples (20 mL) through a pre-weighed, dry GF/F Whatman glass microfiber filter (Sigma-Aldrich, WHA1825047). After drying the filters at 105 °C for 2 h, TSS was determined from the weight difference of the filter before and after drying.

*Protein Concentration.* Protein levels in extracts were measured using the Pierce Bicinchoninic Acid (BCA) Protein Assay kit (Thermo Fisher, 23225), adhering to the protocol provided by the manufacturer. In summary, 150 µL of enzyme extract or protein standard solution (bovine serum albumin, final protein concentration range: 0 - 400 µg/mL) was added to 150 µL of working reagent in a transparent 96-well plate. The plate was incubated for two hours at room temperature in the dark and absorbance was measured at 562 nm using a Tecan Infinite 200 pro plate reader.

*Peptidase Activity*. Peptidase activity was determined using the EnzChek Protease Assay kit (Thermo Fisher, E6638), which employs fluorogenic casein as substrate. The working solution was prepared following the manufacturer’s guidelines. 100 µL of enzyme extract was combined with 100 µL working solution in a well of a black 96-well microplate (Eppendorf, Microplate 96/U-PP, black wells). The formation of fluorescent hydrolysis products was monitored over 80 minutes using a Tecan Infinite 200 pro plate reader, set to an excitation wavelength of 485 nm and an emission wavelength of 530 nm, recording measurements every 4 minutes. The increase in fluorescence intensity within 60 minutes was used for peptidase activity quantification.

**UHPLC-HRMS/MS Measurements**

We analysed the peptides using UHPLC (Thermo Scientific Vanquish Horizon) equipped with a Waters XSelect PREMIER CSH C18 column (article number: 186009870) coupled to HRMS (Thermo Exploris 240). We injected 0.5 µg peptide (5 µL) from each sample and applied a flow rate of 300 µL/min using the following eluent gradient (A: Ultrapure water, B: 95 % LCMS-grade acetonitrile; both containing 0.1 % LCMS-grade formic acid): 0-1 min: 5 % B, 1-14 min: 5 % B – 30 % B, 14-15 min: 30 % B – 100 % B, 15-18 min: 100 % B, 18-18.5 min: 100 % B – 5 % B, 18.5 - 20 min: 5 % B. Detection parameters were chosen as follows: MS full-scan: range: 150 – 2000 m/z, resolution: 120’000, AGC target: Standard, Maximum IT: Auto, positive electrospray ionization (tune data: ion transfer tube temperature: 325 °C, sheath gas: 40, aux gas: 10, sweep gas: 1, RF Lens: 70.0), MS/MS acquisitions: Top12, resolution: 22’500, AGC target: Standard, Maximum IT: Auto, isolation window: 2.0 m/z, NCE: 30, dynamic exclusion time: 2.0 s. Skyline (version 20.2.0.343) was used for parent peptide degradation analysis. Following criteria for parent peptides and hydrolysis product identification were used^2^: m/z deviation < 2 ppm, MS2 fragments with m/z deviation < 5 ppm, and reasonable chromatographic peak shape.

**Data Processing**

Data processing was performed using Peaks Studio 11 (Bioinformatics Solutions Inc.). The workflow^3^ included label-free quantification and data base search against the peptide sequences of the peptide library. Precursor tolerance was set to 20 ppm and 0.01 Da for MS2 fragments. No protease digestion was specified. A false discovery rate threshold was set to < 1 % for all peptides and peptides with a quality score ≤ 30 % were removed. NormalyzerDE^4^, utilizing a generic retention time segmented approach to mitigate time-resolved bias, was utilized for data evaluation and normalization. Dixon’s Q test was implemented to eliminate outliers from replicate samples.

**Table S1.** Wastewater treatment plant (WWTP) parameters on sampling days. Provided are exact sampling dates (sampling occurred always between 8:00 and 9:30 am), influent volumes, temperatures, and pH values on the respective sampling date. Solid and hydraulic retention times of the respective WWTP and their operational type. Maximal capacities refers to maximum population equivalents that the respective WWTP can treat. AT indicates the high-load aeration tank; Asterisk indicates a 24-hour composite sample where 30 mL water were sampled volume-proportionally every 40 m^3^ of influent load. N.A. indicates non-available data.

| **Parameter** | **WWTP A** | | | | **WWTP B** | | **WWTP C** |
| --- | --- | --- | --- | --- | --- | --- | --- |
| Sampling Location | Influent | Influent* | AT | AT | Influent | AT | Influent & AT |
| Type | Activated sludge | | | | Activated sludge | | Activated sludge |
| Maximal capacity | 55’000 | | | | 4’000’000 | | 30’000 |
| Sampling date | 12.12.23 | 28.02.24 | 29.02.24 | 28.03.24 | 18.12.23 | 04.04.24 | 27.02.24 |
| Influent volume on sampling day (m^3^) | 9’394 | 5’966 | 5’799 | 6’989 | 522’541 | 512’388 | 4'314 |
| Influent Temperature | 12.6 °C | 13.3 °C | 13.5 °C | 13.8 °C | 16.3 °C | 16.9 °C | 13.2 °C |
| Influent pH | N.A. | 7.3 | 7.3 | 7.4 | 7.8 | 7.8 | 8.0 |
| Solid retention time AT | - | | 12 h | 12 h | - | 1 d | 31 d |
| Hydraulic retention time AT | - | | 2.2 h | 2.2 h | - | 2.3 h | 36 h |

**
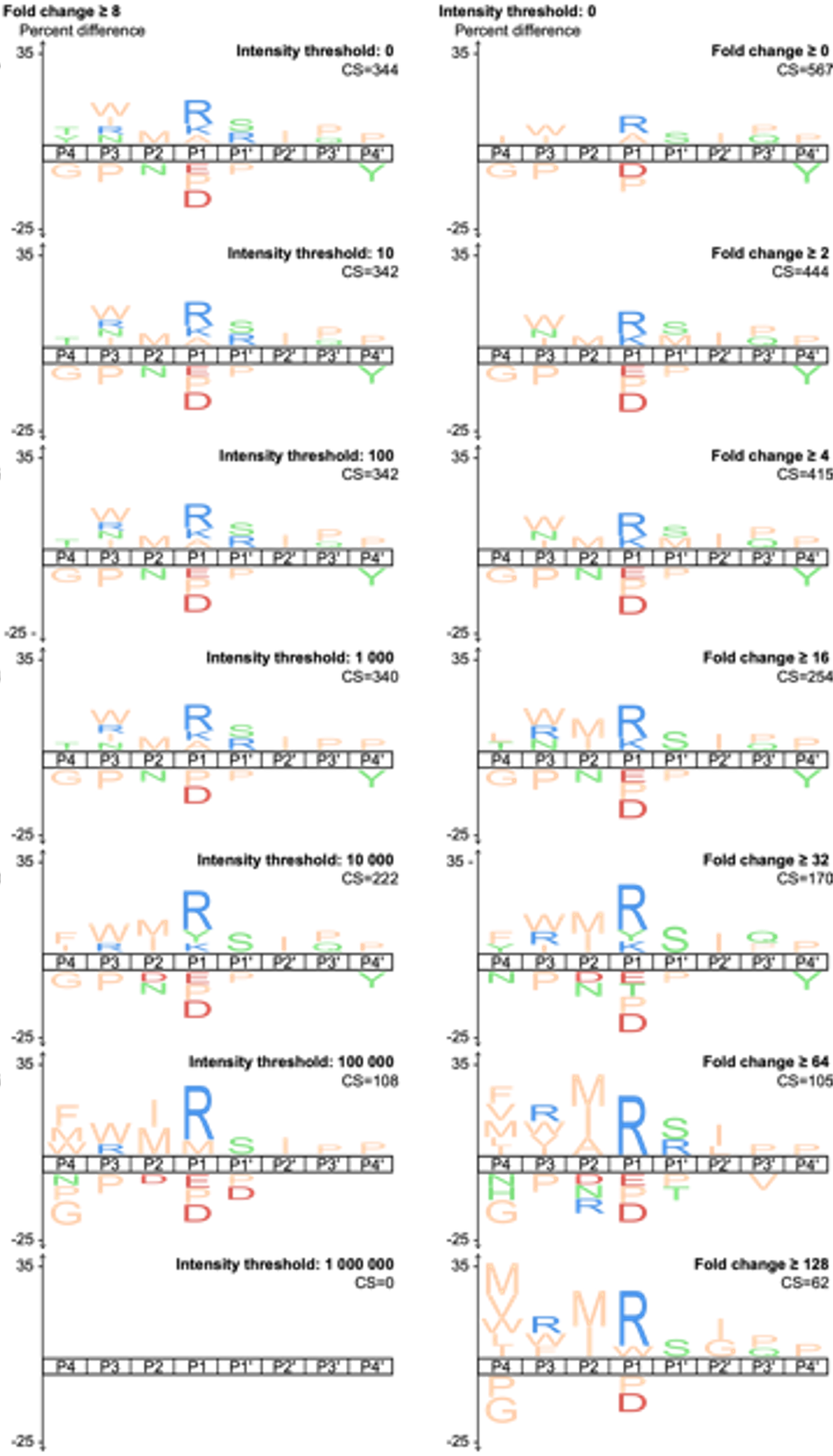
**

**Figure S1.** Effect of different parameter thresholds of the multiplex substrate profiling (MSP-MS) workflow on the specificity profiles. IceLogos represent the amino acid frequency surrounding the detected hydrolysis sites relative to their frequency in the library. The four amino acids on the N-terminal and C-terminal part of the hydrolysis site are referred to as P4-P1 and P1’-P4’, respectively. One letter amino acid code is used with M representing norleucine. CS indicates the number of detected cleavage sites. Blue: Positively charged amino acids. Red: Negatively charged amino acids. Green: Polar amino acids. Orange: Non-polar amino acids. Residue polarity and charge state defined at neutral conditions (pH=7). **Left column**: Different signal intensity thresholds for signal detection in Peaks were used. Transformation products were defined as peptides with peak areas that were eightfold or more increased in the active sample compared to the autoclaved sample. **Right column**: Comparison of different fold changes (i.e., the factor by which the peak area in the active sample exceeded the peak area of the same transformation product in the autoclaved sample) on the MSP-MS results (intensity thresholds were set to 0 for these analyses).


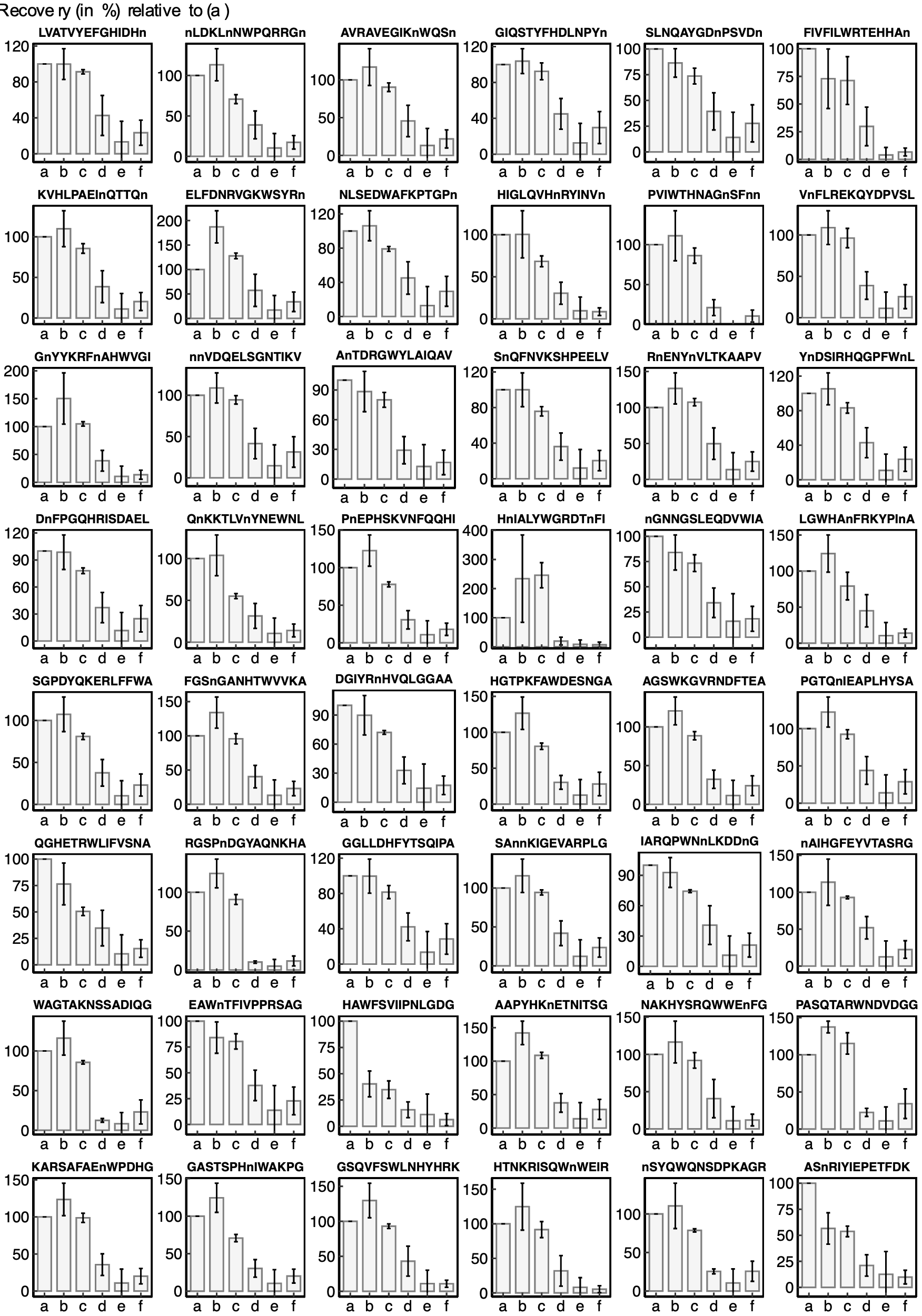


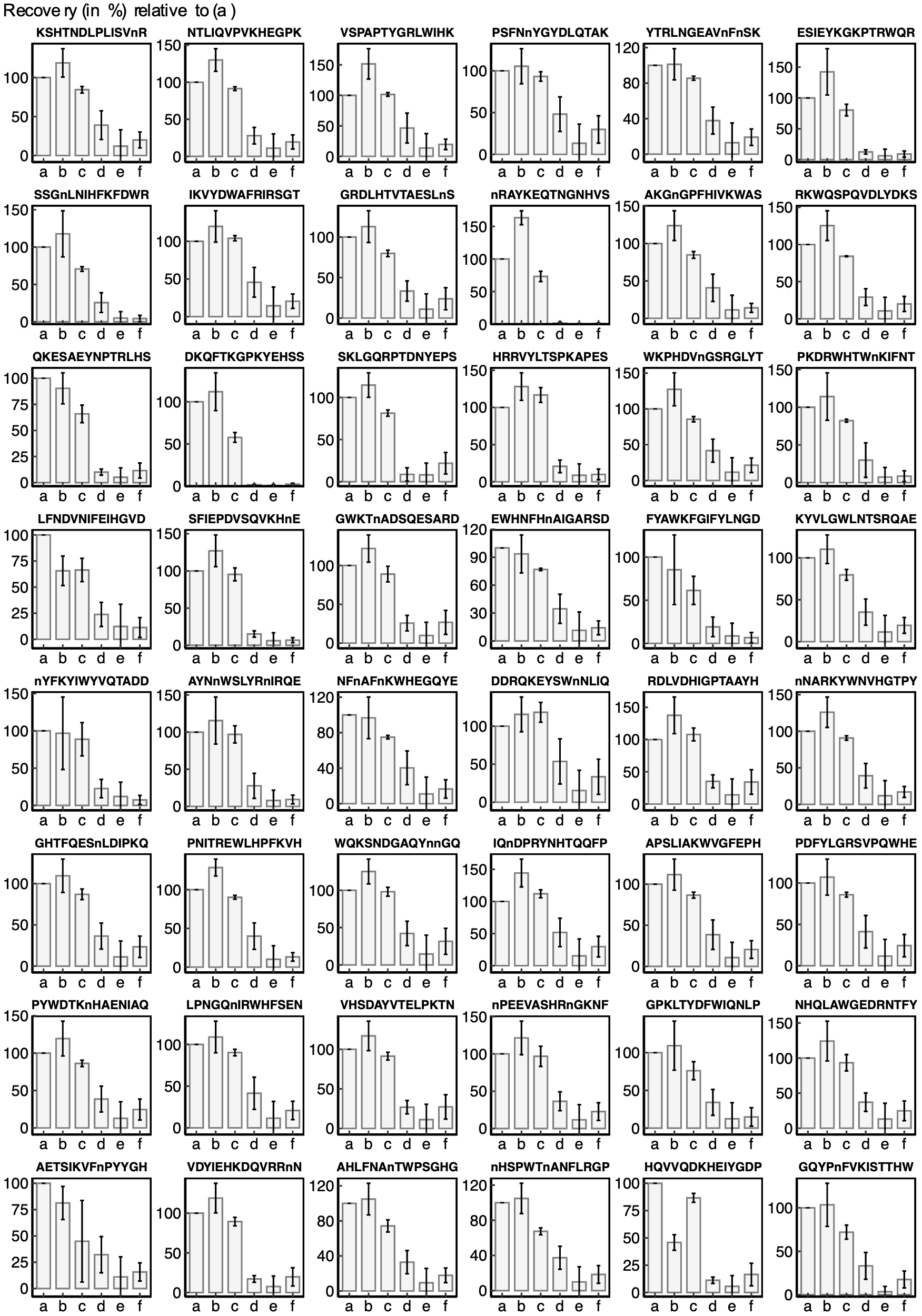


**
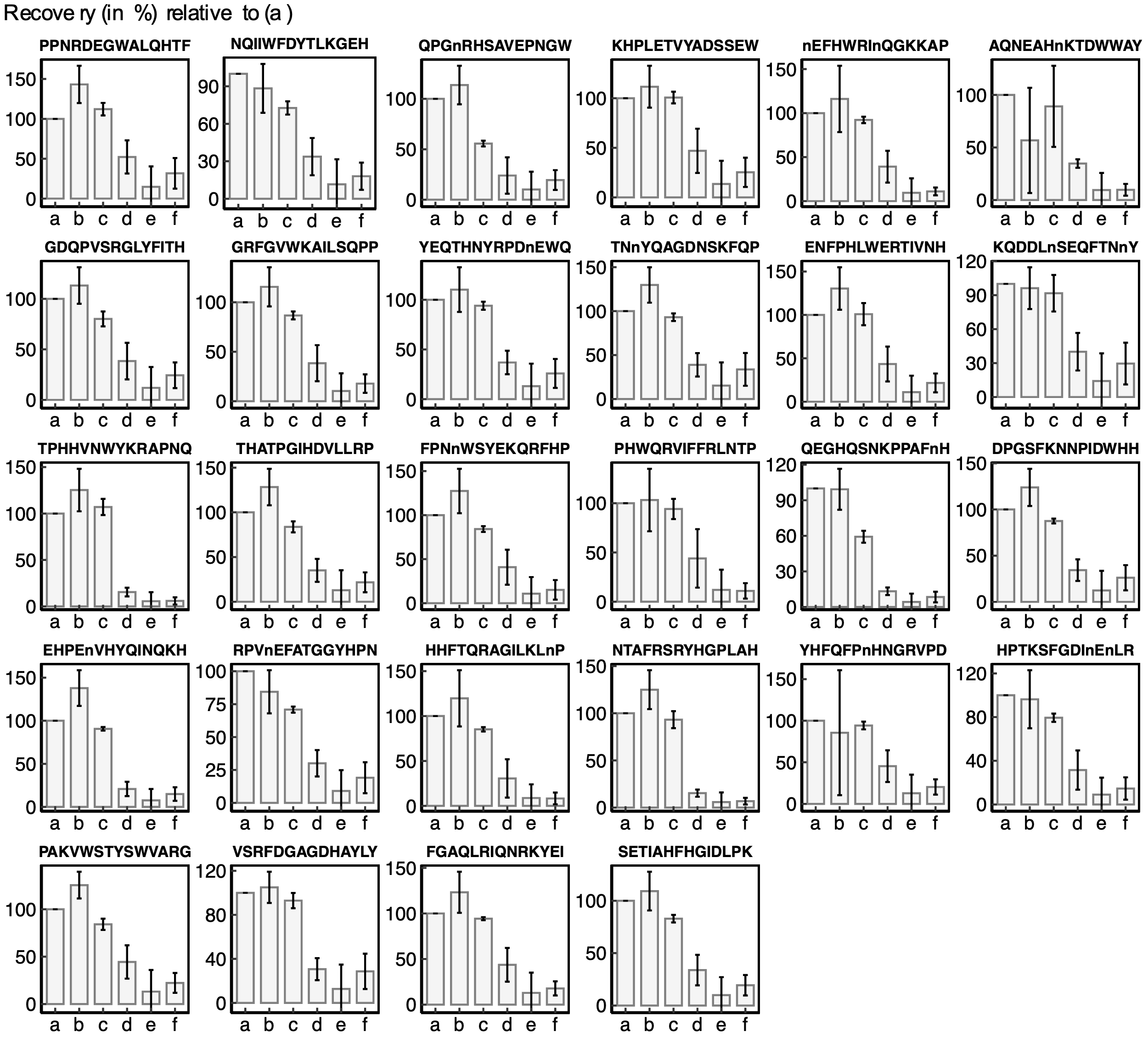
Figure S2.** Comparison of peptide recoveries using different protocols to stop enzymatic activity. Peptides were spiked to ultrapure water to a final concentration of 0.5 µM per peptide. Sequences above bar plots indicate the amino acid sequence of the respective peptide using one letter amino acid code with n representing norleucine. **a**: No treatment. **b**: Heating samples to 90 °C for 10 minutes, centrifugation, transfer of supernatant to LCMS vials. **c**: Samples precipitated in acetonitrile containing 1 % formic acid, centrifugation, transfer of supernatant and evaporation of solvent prior to resuspension and transfer to LCMS vials. **d**: Acidification of samples using 1 % formic acid, centrifugation and transfer of supernatant to LCMS vials. **e**: Samples added to 8 M urea, followed by solid phase extraction using C18 ZipTip columns. **f**: Solid phase extraction using C18 ZipTip columns (without urea treatment). Data points and error bars represent means +/- standard deviations of triplicate incubations (except for a, which were single measurements).


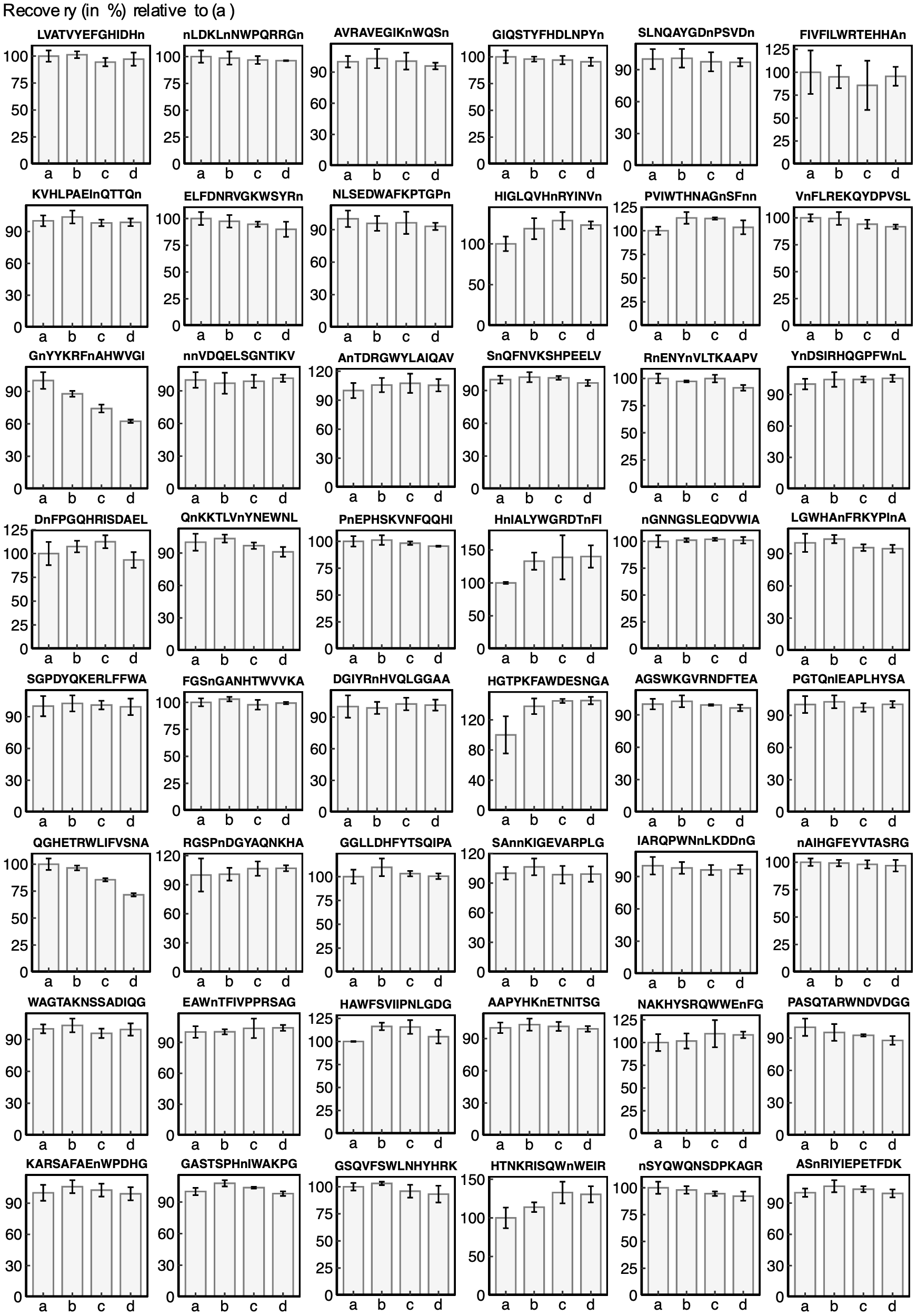


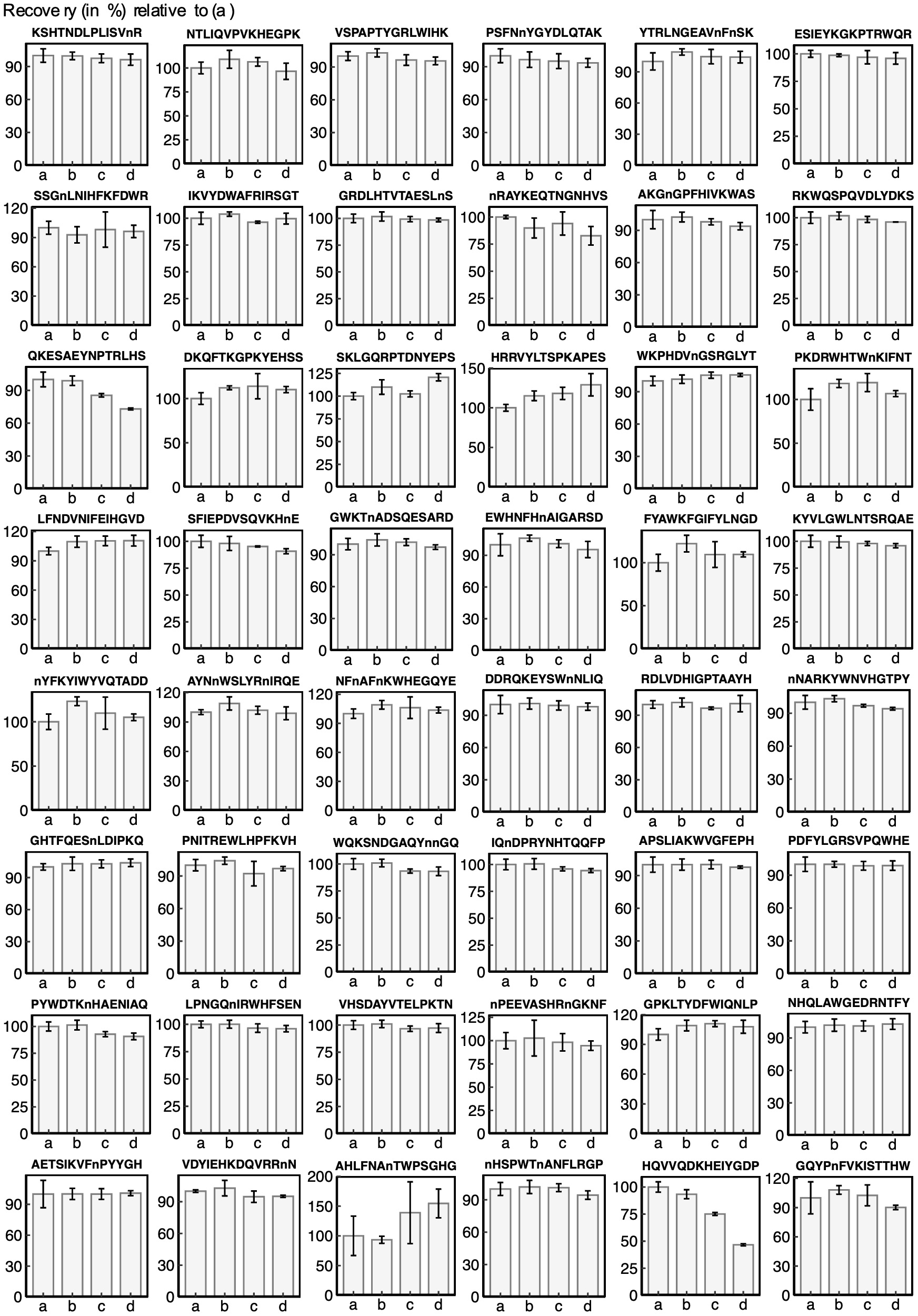


**
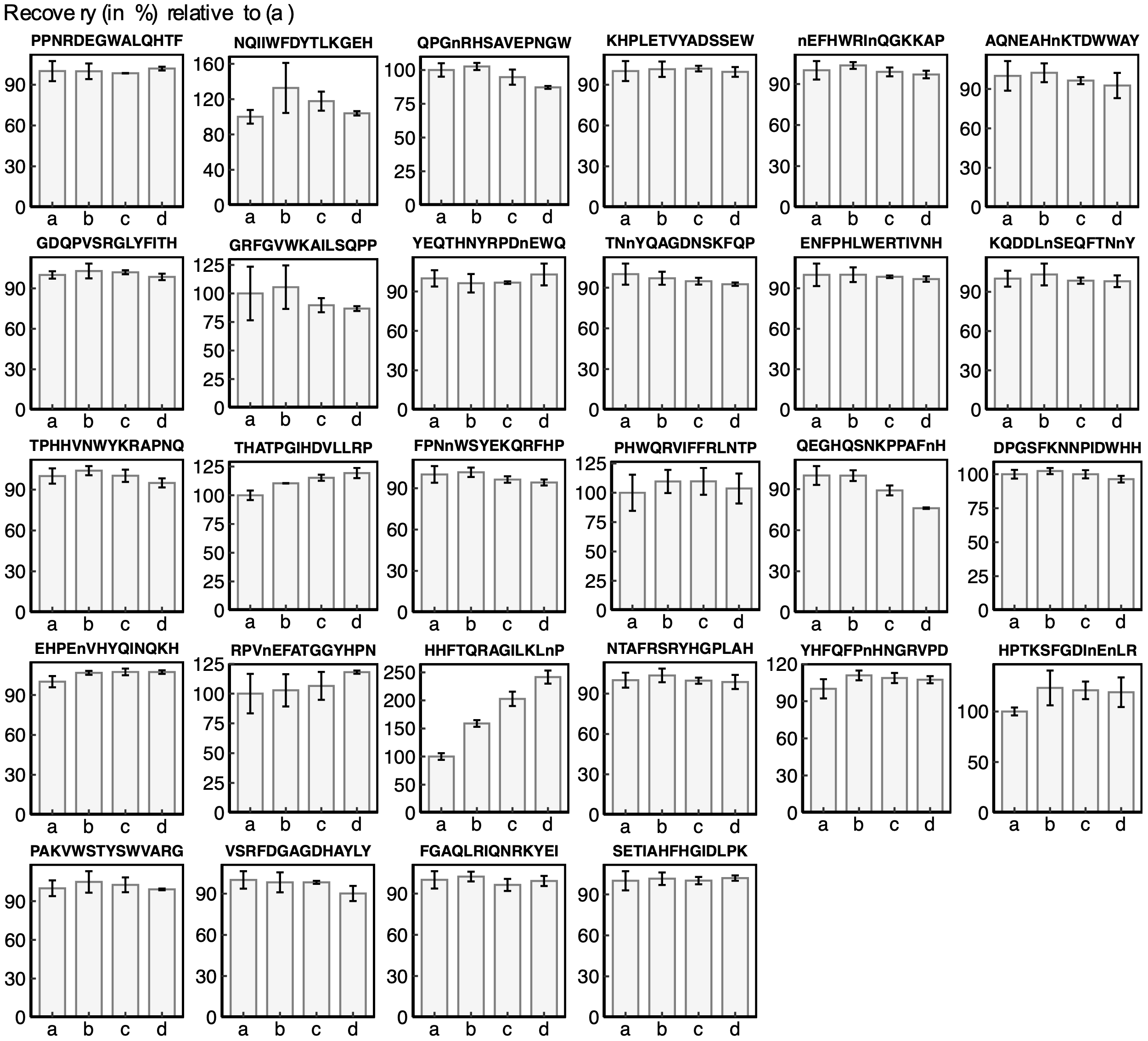
Figure S3.** Comparison of peptide recoveries using different temperatures to stop enzymatic activity. Peptides were spiked to ultrapure water to a final concentration of 0.5 µM per peptide. Sequences above bar plots indicate the amino acid sequence of the respective peptide using one letter amino acid code with n representing norleucine. **a**: No treatment. **b**: Heating samples to 70 °C for 10 minutes, centrifugation, transfer of supernatant to LCMS vials. **c**: Heating samples to 80 °C for 10 minutes, centrifugation, transfer of supernatant to LCMS vials. **d**: Heating samples to 90 °C for 10 minutes, centrifugation, transfer of supernatant to LCMS vials. Data points and error bars represent means +/- standard deviations of triplicate incubations.


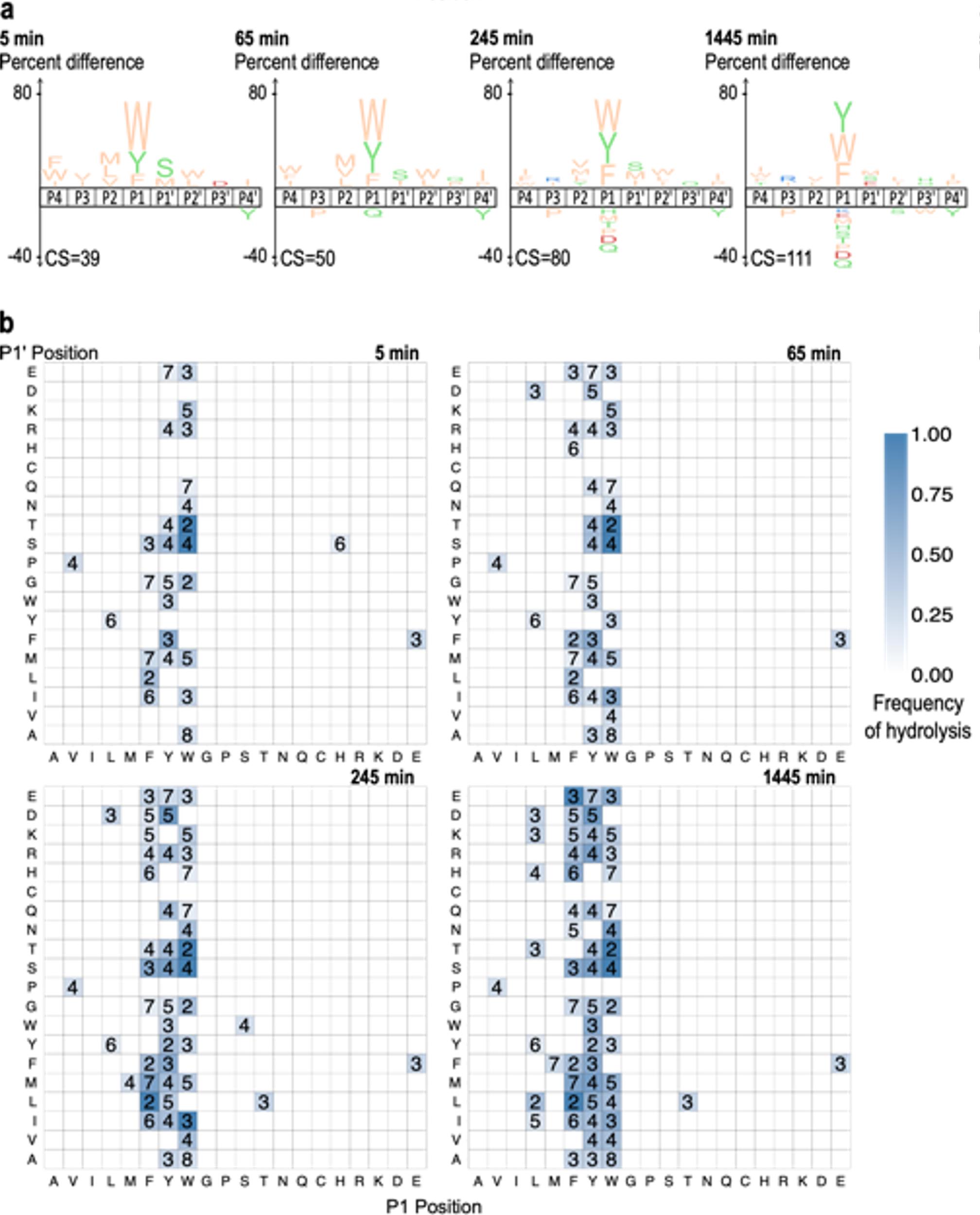


**Figure S4.** Multiplex substrate profiling by mass spectrometry workflow validation using α-chymotrypsin. **a**: Specificity profiles of incubations with α-chymotrypsin after different time points of incubation. IceLogos represent the amino acid frequency surrounding the detected hydrolysis sites relative to their frequency in the library. The four amino acids on the N-terminal and C-terminal part of the hydrolysis site are referred to as P4-P1 and P1’-P4’, respectively. One letter amino acid code is used with M representing norleucine. CS indicates the number of detected cleavage sites. Blue: Positively charged amino acids. Red: Negatively charged amino acids. Green: Polar amino acids. Orange: Non-polar amino acids. Residue polarity and charge state defined at neutral conditions (pH=7). **b**: Amino acid pairs (P1-P1’) flanking the hydrolyzed peptide bond for α-chymotrypsin incubations after distinct time points of incubation. One letter amino acid code is used with M representing norleucine. The number in each cell represents the occurrence of the amino acid pair in the peptide library; the cell color represents the relative hydrolysis frequency. The absence of an amino acid pair in the heatmap does not necessarily mean that the bond was not hydrolyzed, but could also indicate that the product was not detectable or that the product was further hydrolyzed.


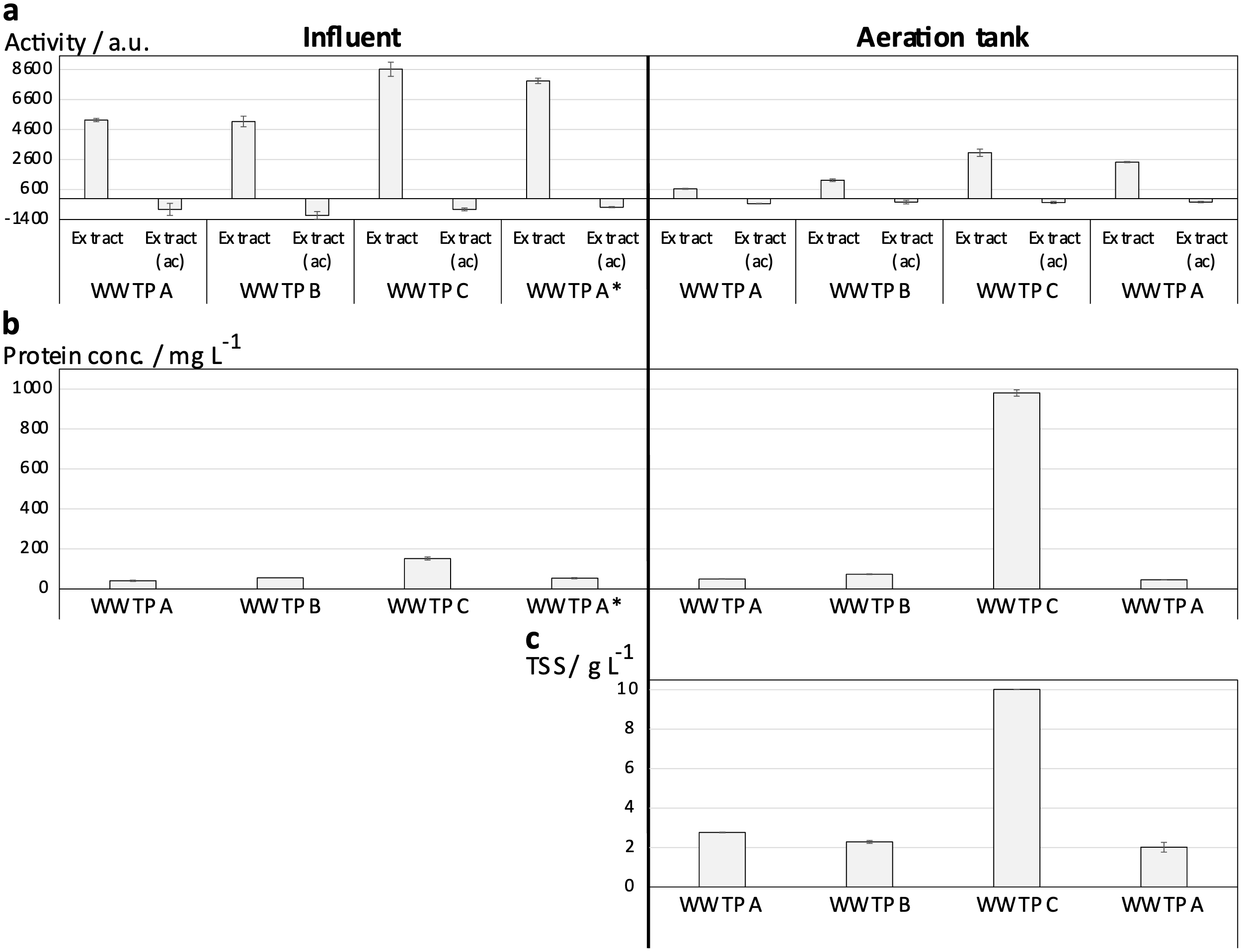


**Figure S5.** Peptidase activity and protein concentration of wastewater extracts and total suspended solid concentrations of aeration tank grab samples from three different wastewater treatment plants (WWTPs). **a**: Peptidase activities of dissolved extracellular extracts from influent samples and extracellular dissolved and extrapolymeric substance-bound extracts from aeration tank samples before and after autoclaving (ac). WWTP A* indicates a daily composite sample. Peptidase activity assay based on fluorogenic Casein as substrate. **b**: Protein concentrations of active extracts that are shown in (a). **c**: Total suspended solid concentrations of aeration tank grab samples. Data points and error bars represent means +/- standard deviations of triplicate reactions.

**
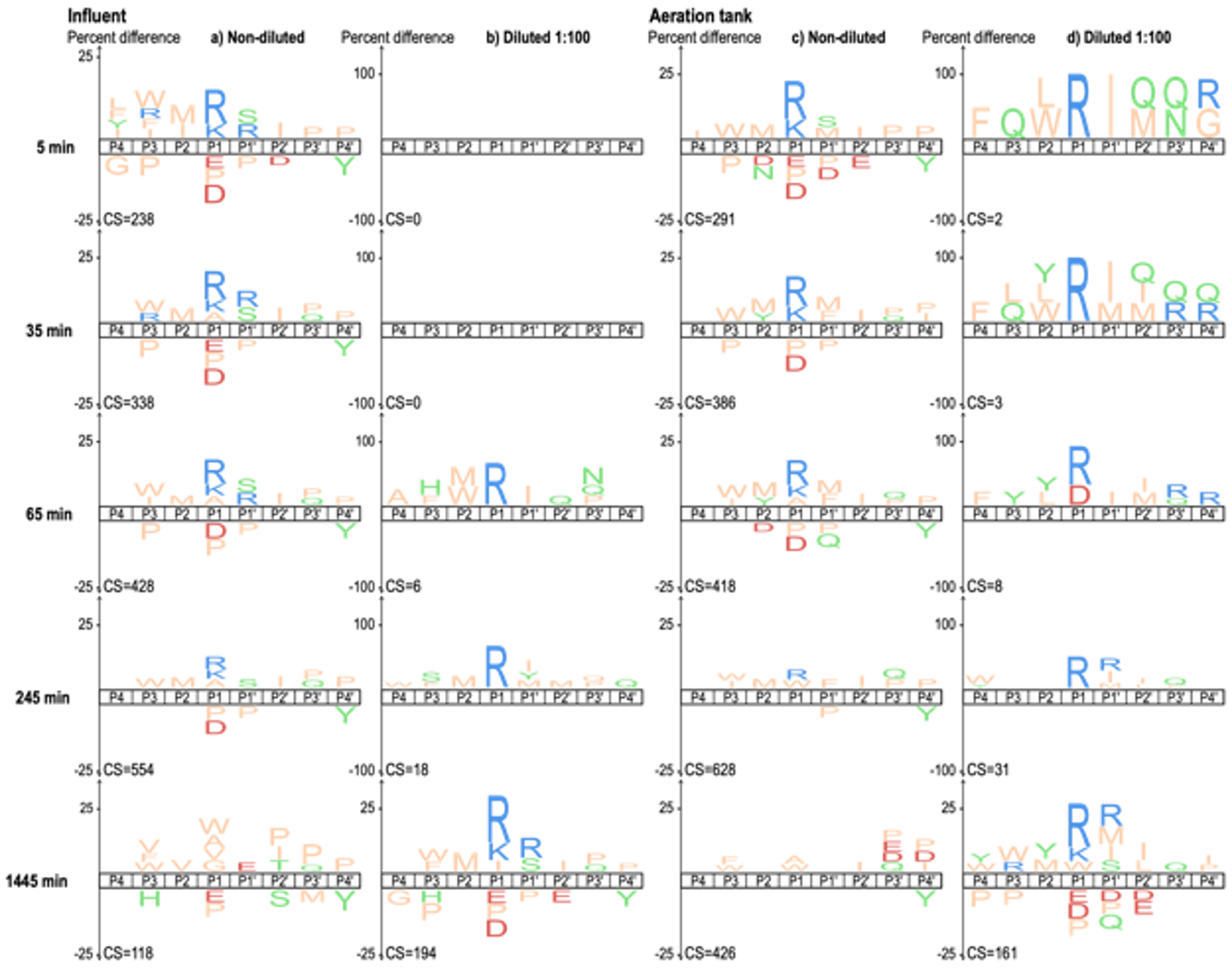
**

**Figure S6.** Specificity profiles of incubations using diluted and non-diluted wastewater extracts with library peptides. **a**: Incubation experiment using non-diluted extracellular dissolved wastewater extract from the influent of WWTP A. **b**: Incubation experiment using extracellular dissolved wastewater extract from the influent of WWTP A diluted 1:100 with autoclaved extract. **c**: Incubation experiment using extracellular dissolved and EPS-bound extract from the aeration tank of WWTP A. **d**: Incubation experiment using extracellular dissolved and EPS-bound extract from the aeration tank of WWTP A diluted 1:100 with autoclaved extract. IceLogos represent the amino acid frequency surrounding the detected hydrolysis sites relative to their frequency in the library. The four amino acid residues located N-terminally and C-terminally of the hydrolysis site are referred to as P4-P1 and P1’-P4’, respectively. One letter amino acid code is used with M representing norleucine. CS indicates the number of detected cleavage sites. Blue: Positively charged amino acids. Red: Negatively charged amino acids. Green: Polar amino acids. Orange: Non-polar amino acids. Residue polarity and charge state defined at neutral conditions (pH=7). Only transformation products with an eightfold increased signal intensity score in the active sample compared to the autoclaved control are considered. Data based on triplicate incubations in active and autoclaved wastewater extracts. Data analysis in Peaks 12.

**
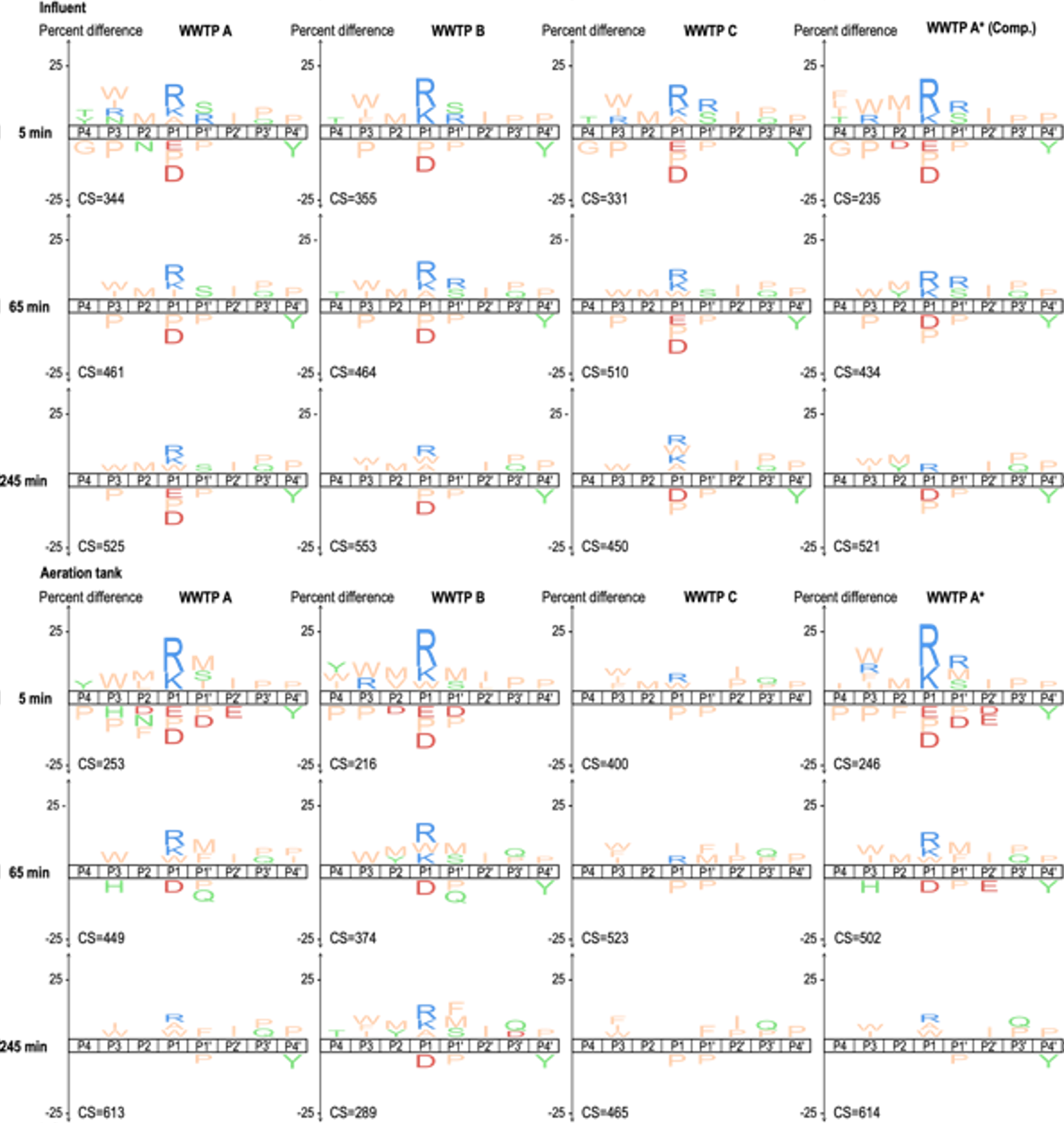
**

**Figure S7.** Specificity profiles of incubations using wastewater extracts with library peptides. IceLogos represent the amino acid frequency surrounding the detected hydrolysis sites relative to their frequency in the library. The four amino acid residues located N-terminally and C-terminally of the hydrolysis site are referred to as P4-P1 and P1’-P4’, respectively. One letter amino acid code is used with M representing norleucine. CS indicates the number of detected cleavage sites. Blue: Positively charged amino acids. Red: Negatively charged amino acids. Green: Polar amino acids. Orange: Non-polar amino acids. Residue polarity and charge state defined at neutral conditions (pH=7). Only transformation products with an eightfold increased signal intensity score in the active sample compared to the autoclaved control are considered. Asterisks indicate samples that were incubated using a slightly different protocol (i.e., one tenth of the entire incubation volume was ultrapure water). For WWTP A* (Comp.), a 24-hour composite influent sample was used. Data based on triplicate incubations in active and autoclaved wastewater extracts.


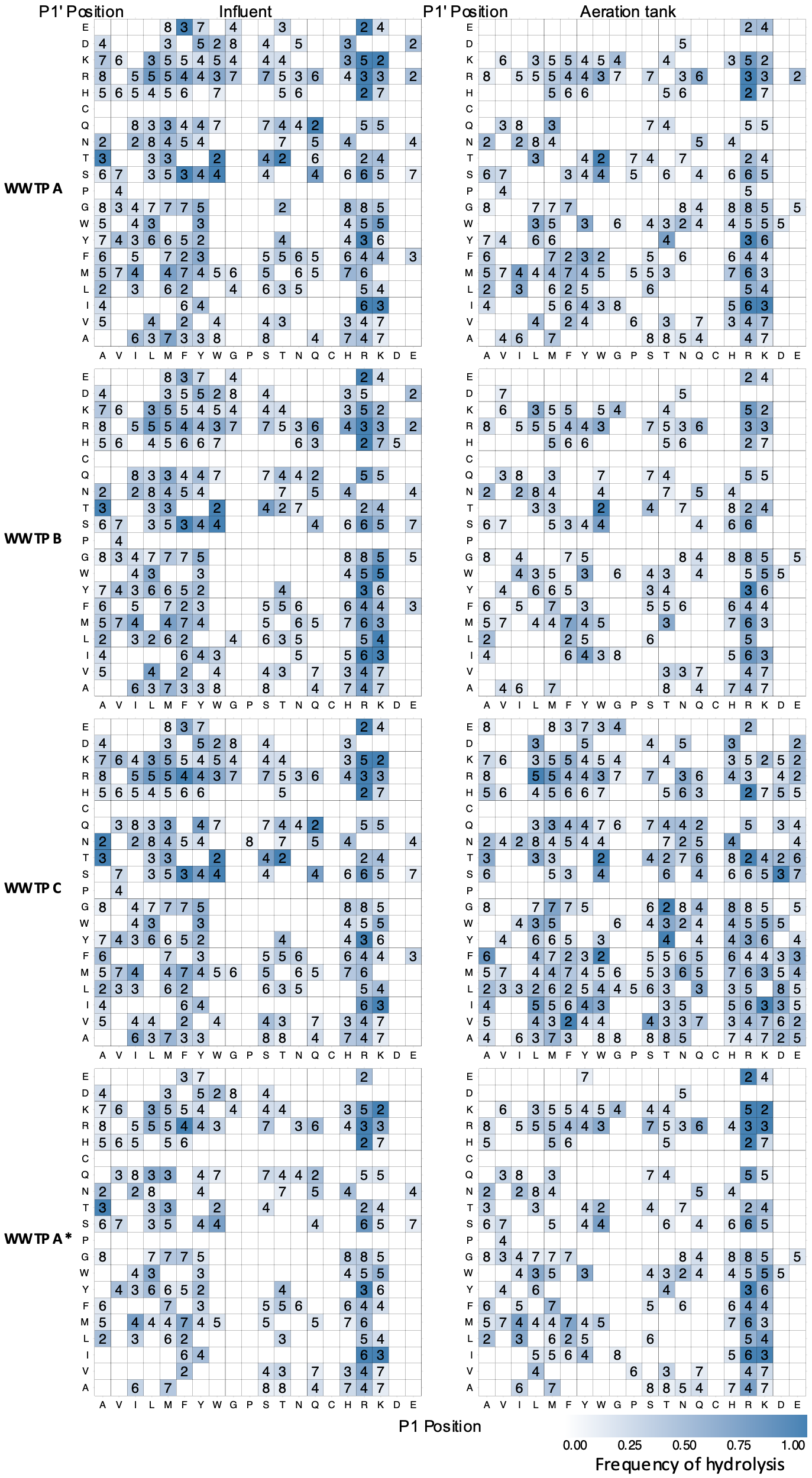


**Figure S8**. Hydrolyzed amino acid pairs during the incubation of wastewater extracts with the library peptides. Amino acid pairs (P1-P1’) flanking the hydrolyzed peptide bond for extracellular dissolved wastewater extracts from influent samples (top) and extracellular dissolved and extrapolymeric substance-bound wastewater extracts from aeration tank samples (bottom) of three full-scale wastewater treatment plants (WWTPs) at the initial sampling time point (t = 5 min). One letter amino acid code is used with M representing norleucine. Asterisks indicate samples that were incubated using a slightly different protocol (i.e., one tenth of the entire incubation volume was ultrapure water). For WWTP A* Influent, a 24-hour composite influent sample was used. The number in each cell represents the occurrence of the amino acid pair in the peptide library; the cell colors represents the relative hydrolysis frequency. The absence of an amino acid pair in the heatmap does not necessarily mean that the bond was not hydrolyzed, but could also indicate that the product was not detectable or that the product was further hydrolyzed.

**Table S2.** Pearson Correlation Coefficients of amino acid frequencies surrounding the cleavage sites of library peptides after hydrolysis by peptidases from different full-scale wastewater treatment plants (WWTPs). A pairwise comparison of the position-dependent amino acid frequencies surrounding the observed cleavage sites for the indicated samples was performed. r indicates the Pearson Correlation Coefficient, R^2^ indicates the Coefficient of Determination of the linear regression for the indicated datasets, and p-value indicates the proportion of randomized datasets that produced a correlation coefficient equal or greater than the observed value (based on 1’000’000 randomizations). For WWTP A* influent, a 24-hour composite sample was used.

| **Comparison** | **System** | **WWTP** | **Pearson Correlation Coefficient r** | **Coefficient of Determination R^2^** | **p-value** |
| --- | --- | --- | --- | --- | --- |
| **Within WWTPs** | Influent compared to aeration tank | WWTP A | 0.79 | 0.63 | 1.9e-05 |
|  |  | WWTP B | 0.87 | 0.75 | 3.1e-05 |
|  |  | WWTP C | 0.68 | 0.46 | 3.3e-04 |
| **Across sampling dates** | Influent | WWTP A / WWTP A* | 0.94 | 0.89 | 2.1e-05 |
|  | Aeration tank | WWTP A / WWTP A* | 0.94 | 0.89 | 2.6e-05 |
| **Across WWTPs** | Influent | WWTP A / WWTP B | 0.96 | 0.91 | 2.4e-05 |
|  |  | WWTP A / WWTP C | 0.97 | 0.95 | 2.8e-05 |
|  |  | WWTP B / WWTP C | 0.95 | 0.90 | 2.9e-05 |
|  | Aeration tank | WWTP A / WWTP B | 0.94 | 0.88 | 2.4e-05 |
|  |  | WWTP A / WWTP C | 0.69 | 0.47 | 7.7e-05 |
|  |  | WWTP B / WWTP C | 0.68 | 0.46 | 4.3e-05 |


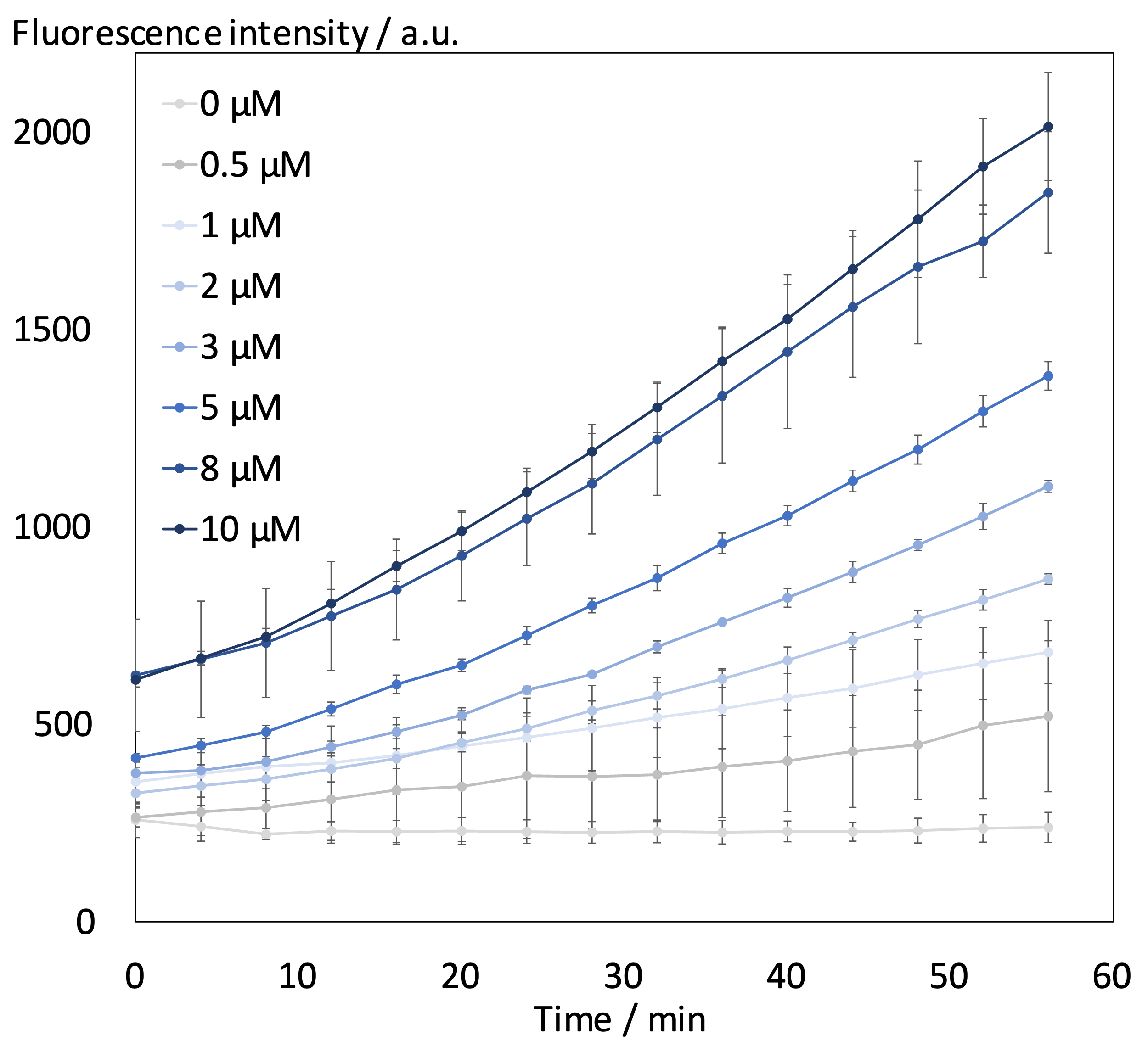


**Figure S9.** Increase in fluorescence intensity upon hydrolysis of a fluorogenic probe by wastewater peptidases. Fluorogenic substrate concentration-dependent increase in fluorescence intensity upon incubation of the fluorogenic probe Leucyl-arginine-4-methylcoumaryl-7-amide (Leu-Arg-AMC) with wastewater extract derived from a full-scale wastewater treatment plant. Each curve represents the incubation of Leu-Arg-AMC at the indicated concentration with wastewater extract. Data points and error bars indicate means +/- standard deviations of triplicate incubations. An AMC calibration was used to derive the substrate conversion for the different substrate concentrations. Linear regression of the curves was performed to determine hydrolysis rates at each substrate concentration.


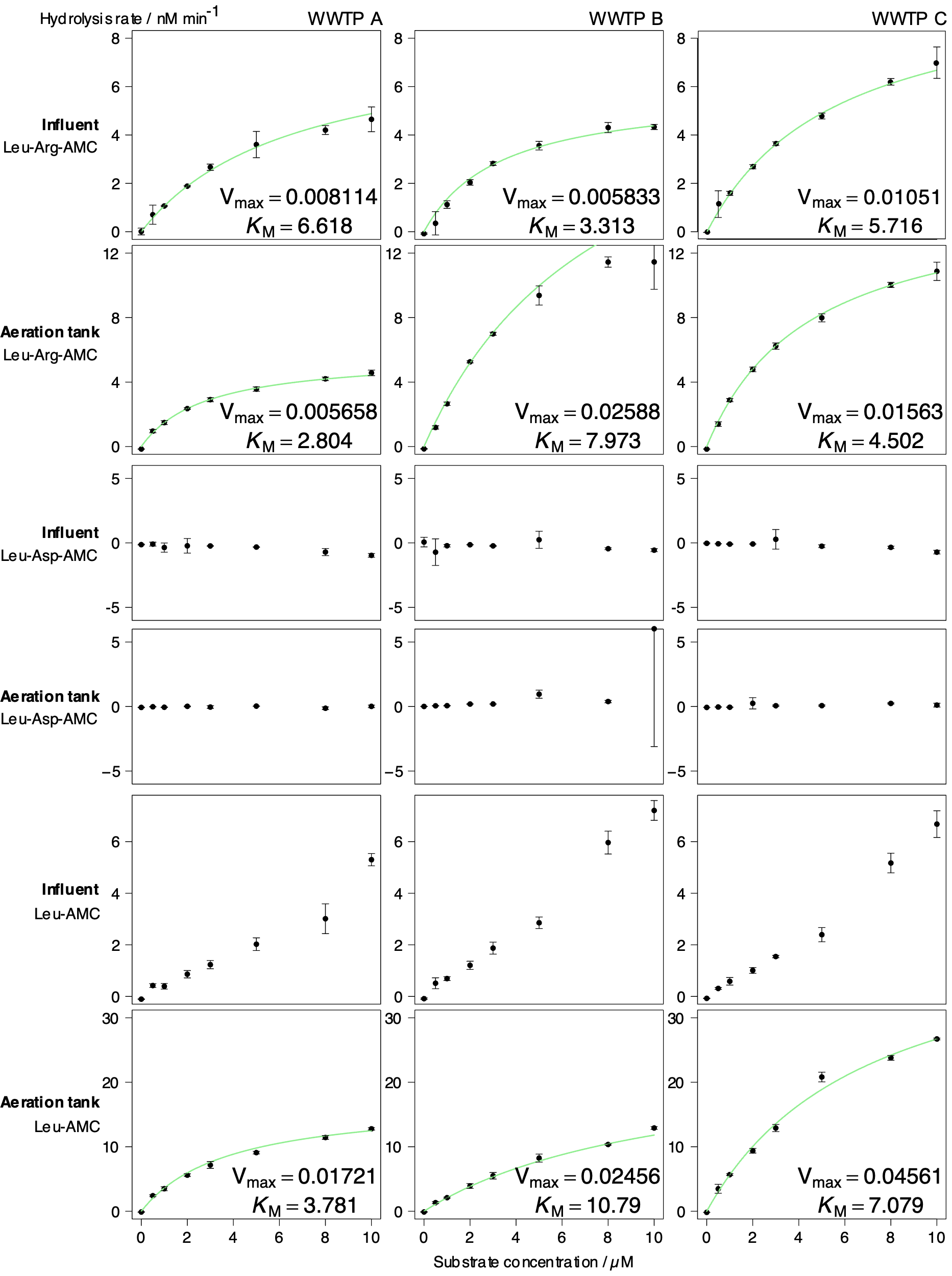


**Figure S10.** Michaelis-Menten reaction kinetics using wastewater extracts incubated with different fluorogenic substrates. Hydrolysis rates derived from slopes of linear increase in fluorescence intensity over time at different substrate concentrations. Data points and error bars represent means +/- standard deviations of triplicate incubations. The fitted curve (green) represents the reaction rate as a function of substrate concentration. For fitting (where applicable), the drm function from the drc R package was used. V_max_ indicates extrapolated maximum reaction velocity; *K*_M_ indicates the Michaelis-Menten constant, which equals substrate concentration at half V_max_. Peptidase substrates represented in three-letter amino acid code: Leucyl-arginine-4-methylcoumaryl-7-amide (Leu-Arg-AMC), Leucyl-aspartic acid-4-methylcoumaryl-7-amide (Leu-Asp-AMC), and Leucine-7-amido-4-methylcoumarine (Leu-AMC).

**References**

1. O’Donoghue, A. J. *et al.* Global identification of peptidase specificity by multiplex substrate profiling. *Nat Methods* **9**, 1095–1100 (2012).

2. Zumstein, M. T. Exploring the Speciﬁcity of Extracellular Wastewater Peptidases to Improve the Design of Sustainable Peptide-Based Antibiotics. *Environmental Science* **54**, 11201–11209 (2020).

3. Rohweder, P. J., Jiang, Z., Hurysz, B. M., O’Donoghue, A. J. & Craik, C. S. Multiplex substrate profiling by mass spectrometry for proteases. in *Methods in Enzymology* vol. 682 375–411 (Elsevier, 2023).

4. Willforss, J., Chawade, A. & Levander, F. NormalyzerDE: Online Tool for Improved Normalization of Omics Expression Data and High-Sensitivity Differential Expression Analysis. *J. Proteome Res.* **18**, 732–740 (2019).
